# Weather parameters and biotic factors synergistically shape the phyllosphere microbiome of pomelo (*Citrus maxima* (Burm.) Merr.) across annual cycle

**DOI:** 10.3389/fpls.2025.1532188

**Published:** 2025-04-03

**Authors:** Weina Yuan, Yongqiang Qin, Wei Zhang, Wenqian Zhou, Guangda Feng, Honghui Zhu, Qing Yao

**Affiliations:** ^1^ Key Laboratory of Biology and Genetic Improvement of Horticultural Crops (South China), Ministry of Agriculture and Rural Affairs, Guangdong Province Key Laboratory of Microbial Signals and Disease Control, College of Horticulture, South China Agricultural University, Guangzhou, China; ^2^ Key Laboratory of Agricultural Microbiomics and Precision Application (MARA), Guangdong Provincial Key Laboratory of Microbial Culture Collection and Application, Key Laboratory of Agricultural Microbiome (MARA), State Key Laboratory of Applied Microbiology Southern China, Institute of Microbiology, Guangdong Academy of Sciences, Guangzhou, China

**Keywords:** phyllosphere microbiome, pomelo (*Citrus maxima* (Burm.) Merr.), annual dynamics, amino acids, weather parameters, leaf chemical traits

## Abstract

Phyllosphere microbiome plays important roles in crop adaptation to the changing environments. Perennial woody crops undergo annual cycles with the changing weather parameters and the biological factors, which might shape the phyllosphere microbial community. In this study, we aimed to investigate the dynamics of phyllosphere microbiome of pomelo (*Citrus maxima* (Burm.) Merr.), an economically important horticultural crops worldwide, and to compare the respective contribution of the weather parameters and the biotic factors to the microbial community assembly, with special focus on the amino acids in leaves. Hi-Seq analysis revealed that both bacterial and fungal communities showed annual cycle dynamics, and the bacterial community in summer was much different from those in other seasons probably due to high temperature and precipitation. However, contribution of the biotic factors (e.g., leaf traits) (12%-29%) to microbial community assembly was higher than that of the weather parameters (4%-15%). Redundancy analysis indicated that the leaf amino acids significantly affected bacterial community while sugars significantly affected fungal community, highlighting the differential patterns of bacterial and fungal community as affected by the biotic factors. Finally, structure equation model showed that the weather parameters influenced microbial community colonizing pomelo leaves both in a direct way and in an indirect way via leaf traits (mainly amino acids). These results demonstrate the primary role of weather parameters and the key role of leaf amino acids in shaping phyllosphere microbiome.

## 1 Introduction

Plants are the most important organisms on the Earth, whose leaves produce organic carbon (photosynthates) from CO_2_ and water, and thus sustain the life on the planet. It is inspiring that the global leaf area has increased by 5.39×10^6^ km^2^ during the years of 2000-2017, reaching 1.71×10^8^ km^2^ (Chen et al., 2019). When plant leaves function as the primary productivity, they meanwhile serve as habitats for diverse microorganisms, which are collectively called phyllosphere microbiome. According Peñuelas and Terradas (2014), up to 10^26^ bacteria occupy the global plant leaves. Despite of its huge population size, phyllosphere microbiome has been less investigated, compared with rhizosphere microbiome which has received intense attention for decades (Zhu et al., 2022). However, increasingly accumulated evidence indicates that phyllosphere microbiome plays significant roles in benefiting plants with respect to stress tolerance, growth promotion, nutrient uptake, and disease suppression (Stone et al., 2018). For example, a 1-aminocyclopropane -1-carboxylate- (or ACC-) deaminase producing bacterial strain isolated from the leaves of tropical yam significantly promoted the plant growth of tomato after its colonization of the phyllosphere (Herpell et al., 2023). Epiphytic and endophytic N_2_-fixers in phyllosphere can contribute greatly to plant N nutrition (Zhu et al., 2023). Considering the necessity of leaf disease control, nutrient supply with foliar spray, and aerial spray of stimulants for stress inhibition in most crops, especially in horticultural crops, it is applausive to apply phyllosphere microbiome-based microbiological technology to achieve sustainable development in agriculture industry.

Since phyllosphere microbime can be helpful in plant growth and development, deep insights into the bacterial or fungal community and their driving force are necessary. It is revealed that phyllosphere bacterial community is highly dynamic, with its composition and structure sensitive to the environments including biotic and abiotic factors (Thapa et al., 2017; Li et al., 2023; Wang et al., 2023; Kong et al., 2024). Among biotic factors, the functional traits of host plants have been intensively studied. Earlier studies focused on the foliar C:N:P stoichiometry, which was demonstrated to affect the phyllosphere nitrogen fixing bacterial community or other functional groups (Martirosyan and Steinberger, 2014; Rico et al., 2014). Thapa et al. (2017) indicated that 83% of the observed variance in phyllosphere microbiome could be assigned to the contents of iron, manganese, and chlorophyll b of leaves. Similarly, Yuan et al. (2023) suggested that the contents of isotope carbon and copper, and the leaf area were the main factors influencing the community structure of phyllosphere microbiome. More recently, by using genome-wide association studies (GWAS), Su et al. (2024) revealed that 4-hydroxycinnamic acid, a compound in the phenylpropanoid biosynthesis pathway and synthesized by a rice gene *OsPAL02*, was the main driver for the enrichment of Pseudomonadales, which was the key taxa maintaining phyllosphere microbiome homeostasis. These results strongly point out the importance of plant identity in shaping phyllosphere microbiome (Li et al., 2023).

Plant functional traits can be mediated by abiotic factors, which thus can further exert significant influences on phyllosphere microbiome. For example, light intensity modulated the phyllosphere bacterial community of garden lettuce by affecting the functional composition of leaves (Kong et al., 2024). By analyzing 16S rRNA gene sequences from 1453 leaf samples across China, Wang et al. (2023) revealed that phyllosphere microbiome was mostly explained by climate and host plant factors, with abiotic environmental cues more important at low latitudes. Meanwhile, a great deal of literature shows the involvement of soil physicochemical properties in regulating phyllosphere microbiome (Jia et al., 2020; Zhou et al., 2021), probably via their influences on plant growth performance and leaf chemical composition (Zhu et al., 2022). Taken together, it seems recognized that abiotic factors regulate phyllosphere nicrobiome in an indirect way by affecting host plant traits.

Our previous study on rhizosphere microbiome found that soil amino acids could profoundly regulate rhizosphere bacterial community (Feng et al., 2021), because amino acids can serve as both carbon source and nitrogen source for these soil organisms. Particularly, application of exogenous phenylalanine enriched functional groups promoting nitrogen cycling and plant growth (Feng et al., 2023a). In contrast, however, the regulation of phyllosphere microbiome by leaf-derived amino acids has not been fully elucidated yet. Moreover, perennial woody plants undergo seasonal changes in the plant traits as affected by the dynamics in weather parameters. Thus, we investigated the annual dynamics of phyllosphere microbiome and plant functional traits of pomelo (*Citrus maxima* (Burm.) Merr.) in this study, which will provide novel insights into the respective contribution of biotic factors and abiotic factors and the regulation of phyllosphere microbiome by amino acids in leaves. We aimed i) to explore the importance of leaf derived amino acids in shaping phyllosphere microbiome, ii) to compare the effects of abiotic environmental cues (weather parameters) and biotic factors on phyllosphere microbiome, and iii) to reveal the annual dynamics of phyllosphere microbiome in pomelo, which will facilitate the rational management and utilization of phyllosphere microbiome for plant growth and health in pomelo.

## 2 Materials and methods

### 2.1 Experimental sites and samplings

The sampling sites in this study are located in Meizhou, Guangdong Province, China, where ‘Sanhong’ pomelo (*Citrus maxima* (Burm.) Merr) is widely planted as cash crops. Leaf samples were taken from two pomelo orchards, namely site 1 (N 24.49207°, E 116.75385°) and site 2 (N 24.35575°, E 116.69304°), across annual cycle spanning four seasons. Briefly, sampling was conducted in Dec. 2022 (winter), Feb. 2023 (spring), May 2023 (summer), and Aug. 2023 (autumn). At each site, nine plants were randomly selected, then four mature and healthy leaves were sampled from each plant. Every twelve leaves from three plants were pooled as one sample, thus producing three biological replicates at each sampling time for each site. The sampled leaves were stored in sterile bags in an icebox and transported to laboratory as quickly as possible. Finally, a total of 24 samples were collected for the analysis of phyllosphere microbiome.

### 2.2 Acquisition of climate data

**Figure 1:**
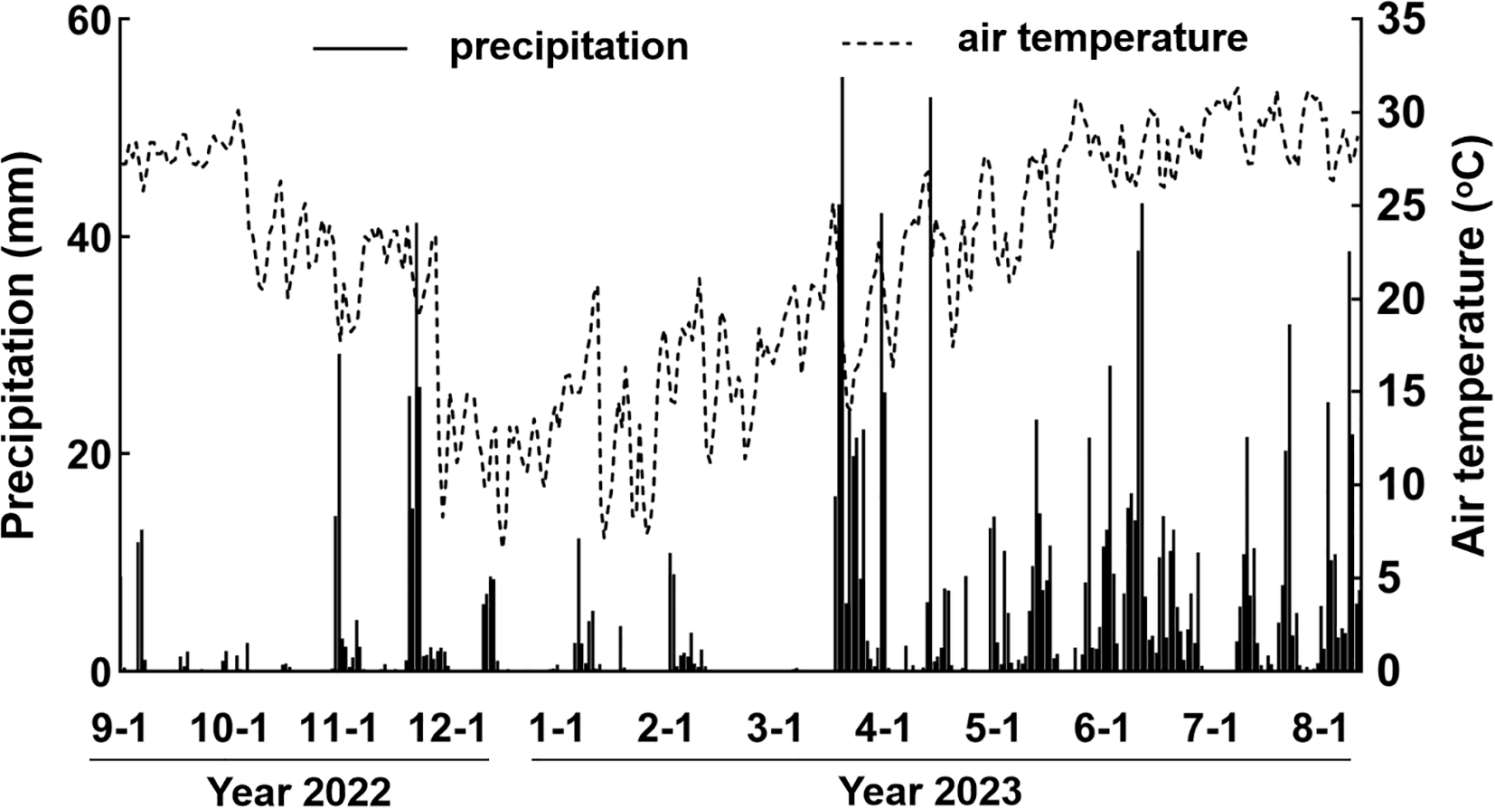
The air temperature and precipitation records during the sampling period.

The sampling sites are typical of subtropical monsoon climate, which is characterized by high temperature (annual average temperature 21.4°C) and concentrated precipitation from Apr. to Sep. (annual average precipitation 1370.26 mm). The climate data (air temperature, precipitation) during the sampling times were retrieved from China Meteorological Data Service Center (http://data.cma.cn/) (**Figure 1**), which was regarded as weather parameters shaping phyllosphere microbiome.

### 2.3 Determination of leaf chemical traits

A total of 24 nutritional constituents were quantified as biological factors (leaf traits) shaping phyllosphere microbiome, which were divided into two categories: amino acid group and non-amino acid group.

Total nitrogen (TN), total phosphorus (TP), and total potassium (TK) in leaves were extracted and quantified according to Belkhodja et al. (1998). N and P contents were determined with the Kjeldahl method and spectro-photometrically, respectively. K content was measured with flame emission spectroscopy. Iron (Fe) content was measured with atomic absorption spectro- photometry. The measurement of sugar contents in leaves was according to Ren et al. (2022) with some modifications. Briefly, fresh samples were ground with liquid N_2_, and then 0.5 g leaf powder was extracted with 10 mL of distilled water at 100°C for 1 h. The extractant was subjected to filtration with 0.22 μm membrane, and the contents of sucrose and fructose were quantified with high pressure liquid chromatography (HPLC). Meanwhile, the contents of NO_3_^-^ and NH_4_^+^ were determined spectro-photometrially at the absorbances of 410 and 625 nm, respectively (da Cunha et al., 2024).

Soluble amino acids in leaves were extracted and determined according to the Chinese National Standard GB/T 30987-2020 (Le et al., 2022). Briefly, 2.0 g fresh leaves were ground with liquid N_2_, incubated with 200 mL boiling water for 30 min, and then filtered with 0.45 µm membrane. The free amino acids in the filtrate were determined using an automatic amino acid analyzer (L-8900, Hitachi) (Feng et al., 2021).

### 2.4 DNA extraction of phyllosphere microorganisms and Hi-Seq analysis

To characterize the phyllosphere (both epiphytic and endophytic) microbiome, the total DNA in leaves (0.25 g) was extracted using the OMEGA E.Z.N.A.^®^ Soil DNA Kit (OMEGA Bio-Tek, Norcross, Georgia, US) according to manufacturer’s instructions. DNA concentration and quality were measured with a NanoDrop 2000 spectrophotometer (Thermo Fisher Scientific, Waltham, MA, United States) and 2% agar-gel electrophoresis.

For the bacterial community, the V5-V6 regions of the 16S rRNA genes were amplified with the specific primers of 799F and 1107R (F: 5′-AACMGGATTAGATACCCKG-3′, R: 5′-GGGTTGCGCTCGTTGCG-3′) (Chen et al., 2022). For the fungal community, ITS1 regions were amplified by PCR with the specific primers of ITS1F and ITS2 (F: 5′-CTTGGTCAT TTAGAGGAAGTAA-3′, R: 5′-GCTGCGTTCTTCATCGATGC-3′) (Li et al., 2022a). The raw image data files obtained by high-throughput sequencing were converted into the original sequence by Base Calling analysis, and the results were stored in the FASTQ file format. It contained the sequence information (Reads) and Reads quality information. Using FLASH software (version 1.2.11) (Magoč and Salzberg, 2011), the Reads of samples were assembled by overlap, and the obtained assembling sequences were the Raw Tags. Using the Trimmomatic software (version 0.3.3) (Bolger et al., 2014), the Raw Tags were filtered to obtain Clean Tags. We obtained the Effective Tags by using UCHIME software (version 8.1) (Caporaso et al., 2010) to identify and remove chimeric sequences. Then, we clustered the Tags to obtain operational taxonomic units (OTUs) at a 97% sequence similarity level by using UCLUST in QIIME (version 1.8.0) (Edgar et al., 2011) and classified OTUs based on the Silva (bacteria) and UNITE (fungi) taxonomic databases.

### 2.5 Bioinformatics analysis and statistics

The ‘vegan’ package was applied to calculate the microbial richness index (observed Chao1, ACE) and diversity index (Shannon-Wiener and Simpson diversity) (Feng et al., 2024). Principal coordinate analysis (PCoA) was performed using the ‘PCoA’ function in ‘ape’ and ‘ggplot2’ packages to visualize the microbial community structure. To determine whether there were significant differences in microbial community structure between seasons, the ‘anosim’ and ‘adonis’ functions in ‘vegan’ package were used for similarity analysis (ANOSIM) and replacement multivariate analysis of variance (PERMANOVA) respectively (Paradis et al., 2004; Wickham, 2011).

The core taxa were defined as the coexistent taxa in four seasons with the relative abundance (RA) >0.1%. Lefse was completed using the Wekemo Bioincloud (https://www.bioincloud.tech). Kruskal-Wallis test (*P*<0.05) and LDA threshold score >2.5 were used to identify biomarkers with significant differences between groups (Gao et al., 2024).

The networks between different subcommunities were analyzed to explore co-occurrence patterns. The Spearman rank coefficient (*ρ*) between OTUs of samples with occurrence rates greater than 50% was calculated using the R package ‘picante’ (Lv et al., 2022) in pairs. Only the robust and significant correlation between OTUs (|*r*|>0.6, *P*<0.05) was selected for network construction. Then, the Gephi (http://gephi.github.io/) was used to visualize the network. In addition, the network topology was calculated in the package ‘igraph’ (Xiong et al., 2018).

The variation partitioning analysis (VPA) was performed using the ‘varpart’ and ‘anova.cca’ functions to measure the contribution of climatic and biological factors to the changes in microbial community structure (Zhao et al., 2020). Random forest (RF) analysis was performed using the ‘RandomForest’ package in R (Shibahara et al., 2017) to determine the importance ranking of each biological factor’s contribution to the difference in alpha diversity indexes between groups. The ‘varclus’ function in the ‘relaimpo’ package was used to test the collinearity of biological factors. Spearman *ρ* ^2^>0.7 indicates that there was collinearity between the biological factors, and one of the representative variables needs to be selected. Redundancy analysis (RDA) was performed using the ‘decorana’ and ‘rda’ functions from the ‘vegan’ package to elucidate the influence of biological factors on the bacterial and fungal community structure (Feng et al., 2023b).

The structural equation model (SEM) in Package R ‘lavaan’ (Tenenhaus et al., 2005) was used to evaluate the effects of climatic and biological factors on microbial community diversity in leaves. The chi-square test, *df* and its associated *P*-values, goods-of-fit index (GFI), approximate root-mean square error (RMSEA), and Akaechi Information criteria (SRMR) were used to determine the fit between the model and the data (good fit when *df*<5, 0.05<*P*≤ 1.00, GFI>0.800, RMSEA ≤ 0.05, SRMR<0.08, lower chisq indicating a better fit) (Feng et al., 2021).

All data were the average of three biological replicates. Multiple range test and *t* test were performed with SPSS v21.0. All the R codes for analysis in this study are available in the following GitHub repository (https://github.com/vn0909/Codes-for-Analysis).

## 3 Results

### 3.1 Phyllosphere microbiome and the core taxa across annual cycle

**Figure 2:**
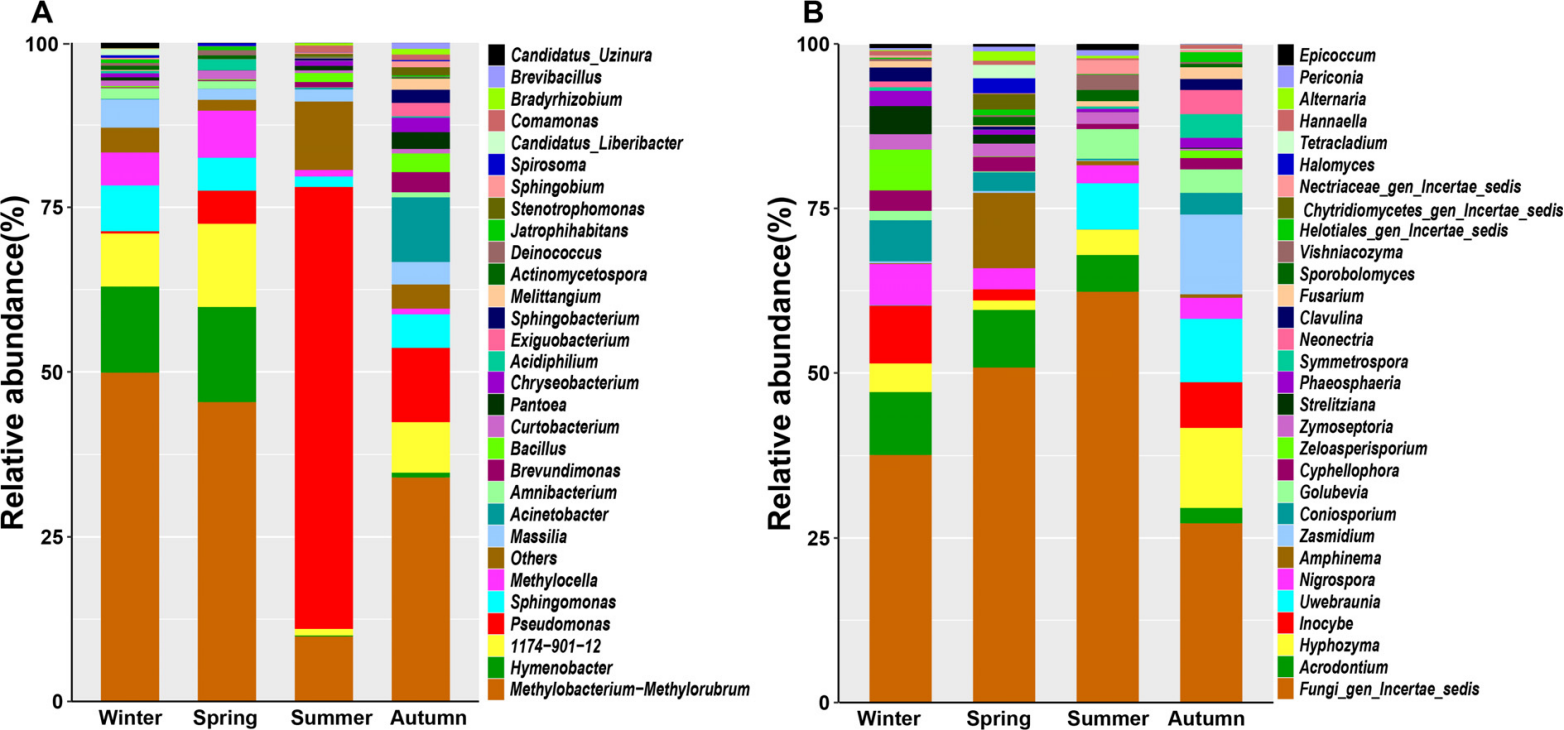
The composition of bacterial **(A)** and fungal **(B)** community at genus level associated with pomelo leaves.

The amplicon sequencing of 16S rRNA and ITS genes revealed diverse bacterial and fungal taxa associated with pomelo leaves. Totally, there were 20 bacterial phyla and 13 fungal phylla, or 58 bacterial genera and 59 fungal genera detected with RA > 0.1% (**Supplementary Data Sheet S1**). The dominant (RA>1.0%) bacterial genera included *Methylobacterium-Methylorubrum*, *Pseudomonas*, *Hymenobacter*, *Sphingomonas*, *Massilia*, *Methylocella*, *Acinetobacter*, *Amnibacterium*, and *Curtobacterium* (**Figure 2A**, **Supplementary Data Sheet S1**), while the dominant fungal genera included *Acrodontium*, *Hyphozyma*, *Inocybe*, *Uwebraunia*, *Nigrospora*, *Amphinema*, *Coniosporium*, *Zasmidium*, *Golubevia*, *Cyphellophora*, *Zeloasperisporium*, *Zymoseptoria*, *Strelitziana*, *Phaeosphaeria*, *Neonectria* (**Figure 2B**, **Supplementary Data Sheet S1**).

**Figure 3:**
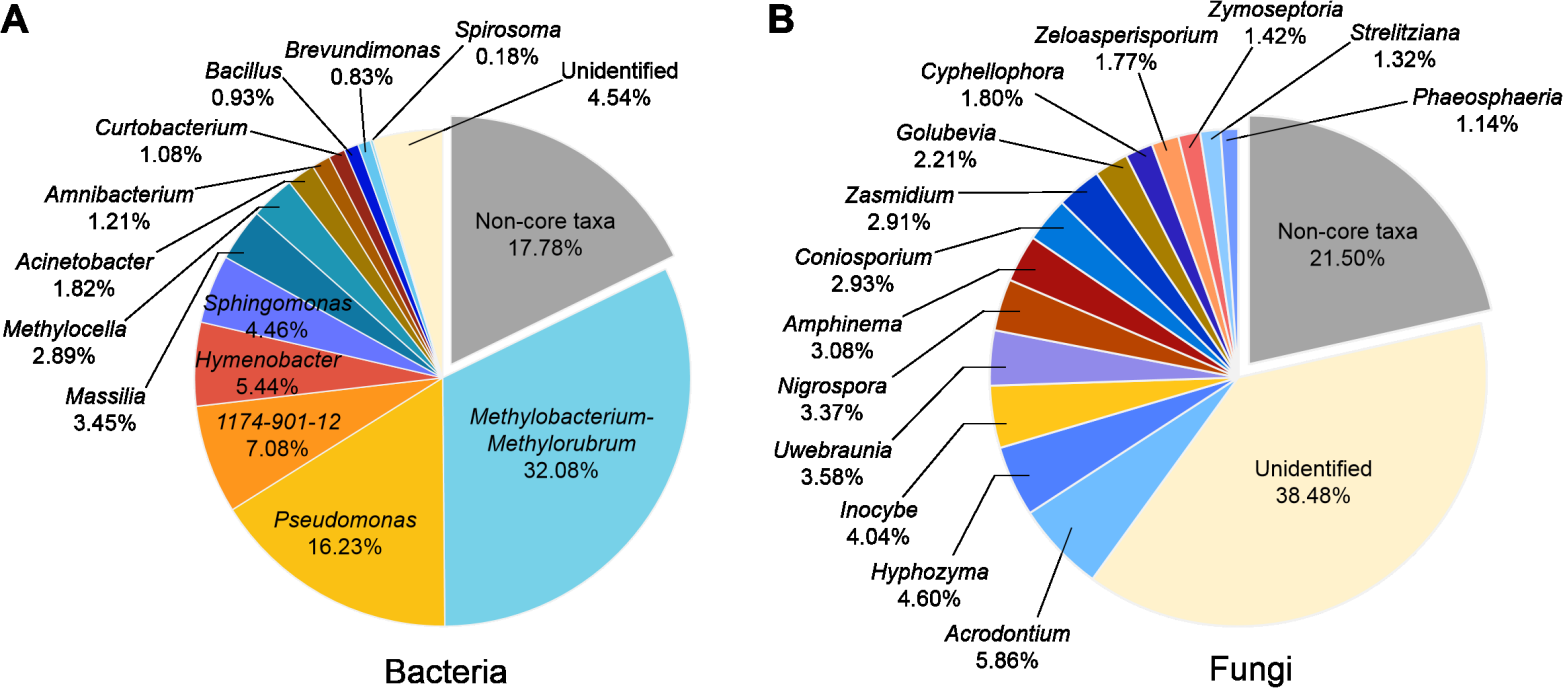
The core genera of bacterial **(A)** and fungal **(B)** community associated with pomelo leaves. The percentages indicate the relative abundance of the responding genus.

On the basis of the occupation and RA, we identified 13 bacterial core genera and 14 fungal core genera in the phyllosphere microbiome. For bacterial core taxa, *Methylobacterium- Methylorubrum* was the most abundant genus (32.08%), followed by *Pseudomonas* (16.23%), 1174_901_12 (7.08%), and *Hymenobacter* (5.44%). These 13 core genera totally occupied 82.32% of the phyllosphere bacterial community (**Figure 3A**). For fungal core taxa, *Acrodontium* was the most abundant genus (RA 5.86%), followed by *Hyphozyma* (4.60%), *Inocybe* (4.04%) and *Uwebraunia* (3.58%). These 14 core genera totally occupied 78.50% of the phyllosphere fungal community (**Figure 3B**).

**Figure 4:**
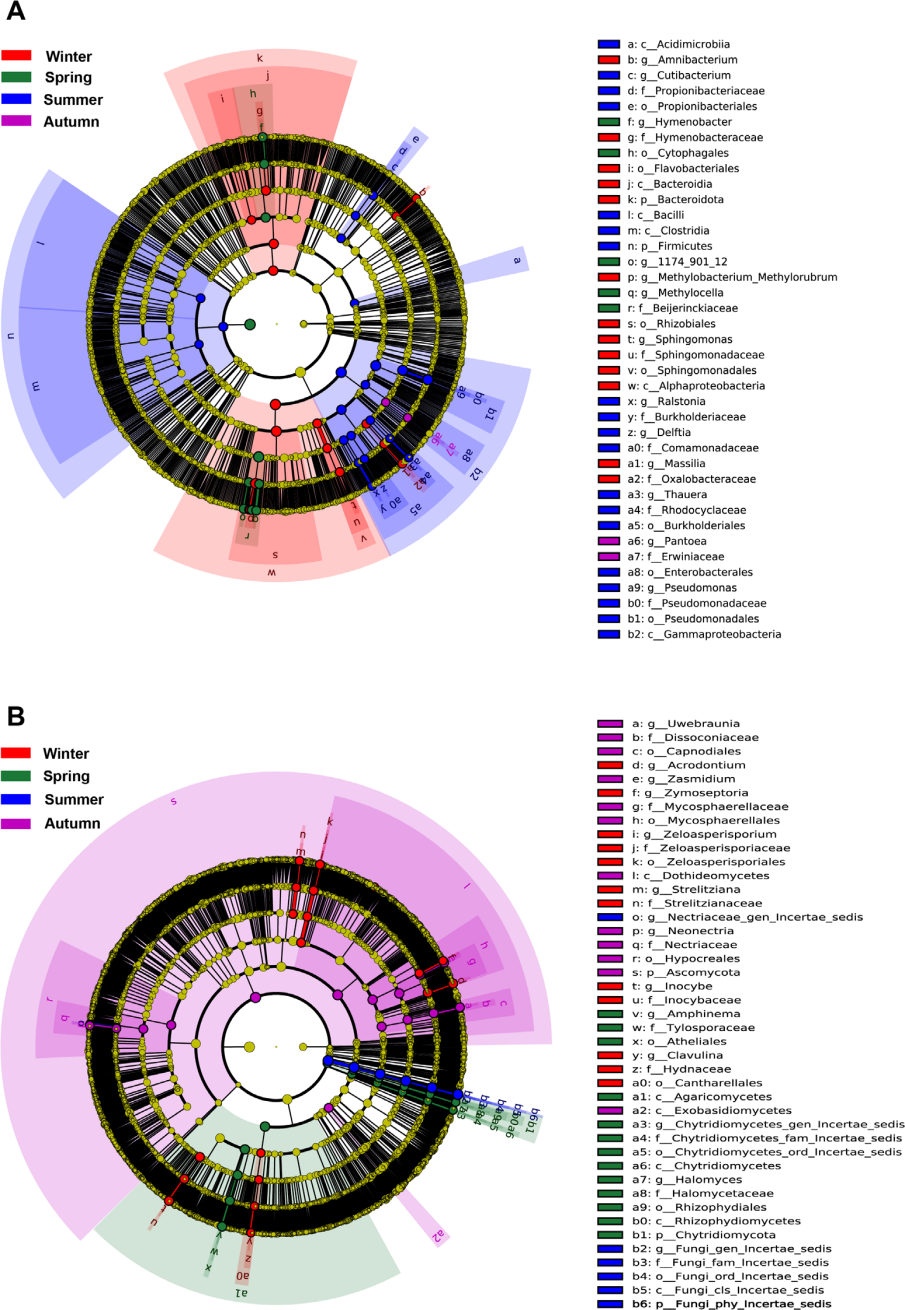
Lefse analysis demonstrating the bacteral **(A)** and fungal **(B)** biomarkers of each season.

In contrast to the core taxa occurring across all seasons, Lefse analysis reveals that there were some specific taxa indicative of each season (**Figure 4**). Four bacterial genera (*Amnibacterium*, *Methylobacterium-Methylorubrum*, *Spingomonas*, *Massilia*) and 5 fungal genera (*Acrodontium*, *Zymoseptoria*, *Zeloasperisporium*, *Strelitziana*, *Inocybe*, *Clavulina*) were significantly enriched in winter. Three bacterial genera (*Hymenobacter*, 1174_901_12, *Methylocella*) and 3 fungal genera (*Amphinema*, *Halomyces*, one unidentified genus) were significantly enriched in spring. Five bacterial genera (*Curtobacterium*, *Ralstonia*, *Delftia*, *Thauera*, *Pseudomonas*) and several unidentified fungal genera were significantly enriched in summer. One bacterial genera (*Pantoea*) and 3 fungal genera (*Uwebraunia*, *Zasmidium*, *Neonectria*) were significantly enriched in autumn (**Figure 4**). It is noteworthy that some core genera were also indicative of particular season, probably suggesting that their seasonal dynamics shaped the phyllosphere microbiome.

### 3.2 Diversity of bacterial and fungal community and their seasonal dynamics

We calculated the alpha diversity of phyllosphere microbiome in different seasons. The highest values of chao1 and richness were observed in winter for bacterial community, and in spring for fungal community. The highest values of shannon diversity were observed in autumn for bacterial community, and in spring for fungal community; but the highest values of simpson diversity were observed in autumn for both bacterial and fungal community (**Table 1**). These data indicate the difference in annual dynamics of bacterial community and fungal community colonizing pomelo leaves.

**Table 1:** The fluctuation of alpha diversity of bacterial and fungal community associated with pomelo leaves across annual cycle.

Sampling time	Chao1	Richness	Shannon	Simpson
Bacterial community				
Winter (Dec. 2022)	596.9 ± 75.35a	483.17 ± 87.45a	3.67 ± 0.48a	0.94 ± 0.03ab
Spring (Feb. 2023)	435.81 ± 46.75b	360.83 ± 43.66b	3.36 ± 0.25a	0.92 ± 0.01ab
Summer (May 2023)	447.46 ± 49.34b	323.5 ± 71.03b	3.36 ± 1.41a	0.77 ± 0.24b
Autumn (Aug. 2023)	438.64 ± 69.64b	387.33 ± 63.91b	3.82 ± 0.13a	0.95 ± 0.00a
Fungal community				
Winter (Dec. 2022)	901.79 ± 94.54b	706.17 ± 62.1b	3.33 ± 0.22a	0.89 ± 0.06a
Spring (Feb. 2023)	1467.68 ± 284.23a	1174.67 ± 243.7a	3.63 ± 0.3a	0.88 ± 0.03a
Summer (May 2023)	1390.5 ± 206.09a	1102.17 ± 194.82a	3.2 ± 0.22a	0.81 ± 0.04b
Autumn (Aug. 2023)	797.31 ± 103.33b	639.33 ± 106.6b	3.32 ± 0.43a	0.91 ± 0.04a

Different letters in each column indicate the significant difference among four seasons according to multiple range test (*P*<0.05, Tukey’s).

**Figure 5:**
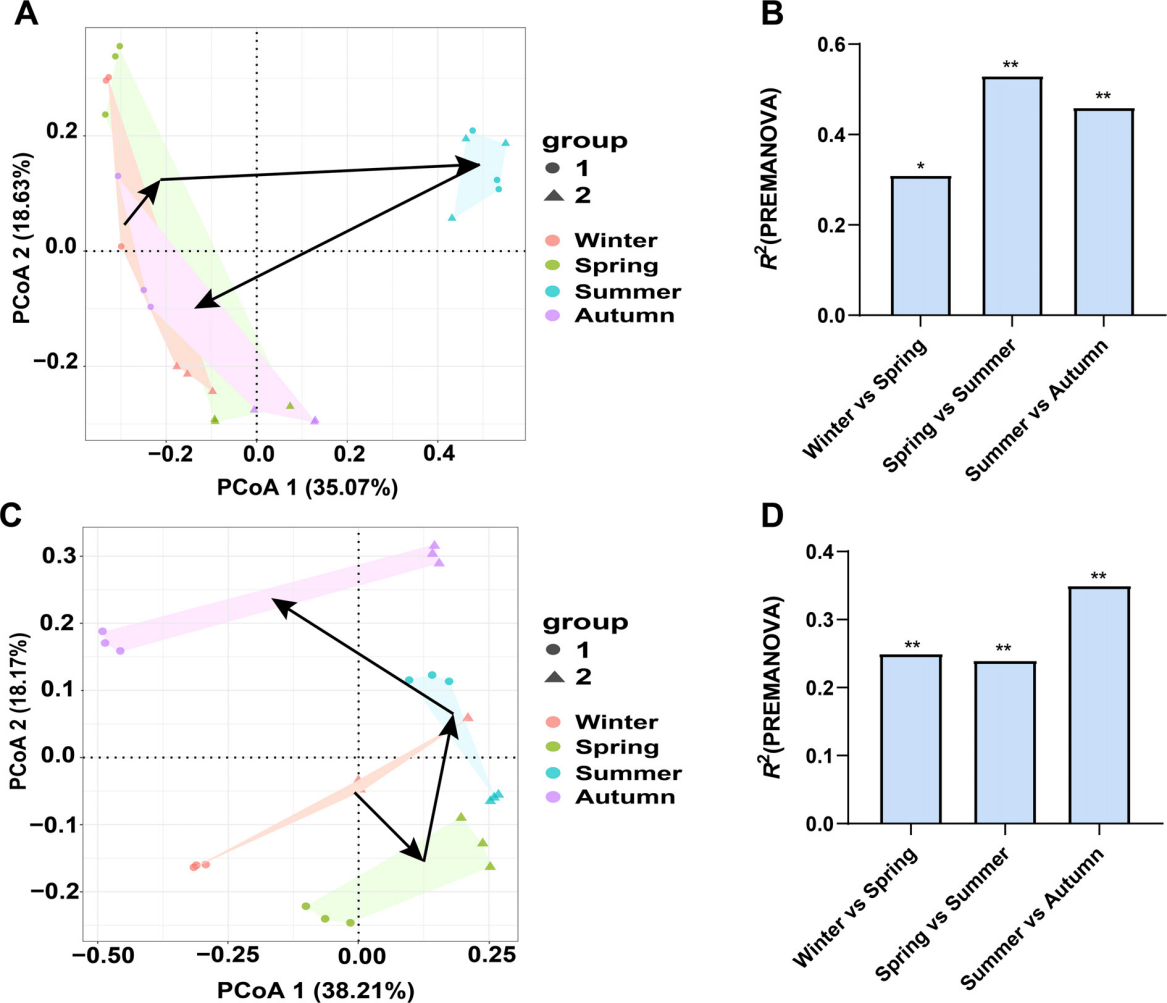
The annual dynamics of bacterial **(A, B)** and fungal **(C, D)** community associated with pomelo leaves. PCoA plotting **(A, C)** demonstrates the difference of microbial community in each season, while PREMANOVA **(B, D)** quantifies the difference in microbial community of two successive seasons. Group 1 and 2 in PCoA plotting **(A, C)** indicate the sampled orchards at site 1 and 2, respectively. * and ** indicate the significant difference in the microbial community between two paired seasons at *P*<0.05 and *P*<0.01 level.

PCoA clearly demonstrates that the bacterial community shifted from winter (2022) to autumn (2023), with the bacterial community in summer much different from those in other three seasons. We calculated the dissimilarity in bacterial community between two successional seasons, and observed a significant difference in spring-to-summer shift and in summer-to-autumn shift (**Figures 5A, B**). This reflects the distinctness of bacterial community in summer compared to other three seasons. In contrast, phyllosphere fungal community showed a different shifting pattern. PCoA plotting demonstrates that the fungal communities in four seasons were much different from each other, which is also confirmed with the significant dissimilarity of winter-to-spring, spring-to-summer, and summer-to-autumn shift. It seems that the fungal community in autumn was much different from that in other three seasons (**Figures 5C, D**).

### 3.3 Effect sizes of abiotic vs biotic factors and annual fluctuation of phyllosphere microbiome networks

**Figure 6:**
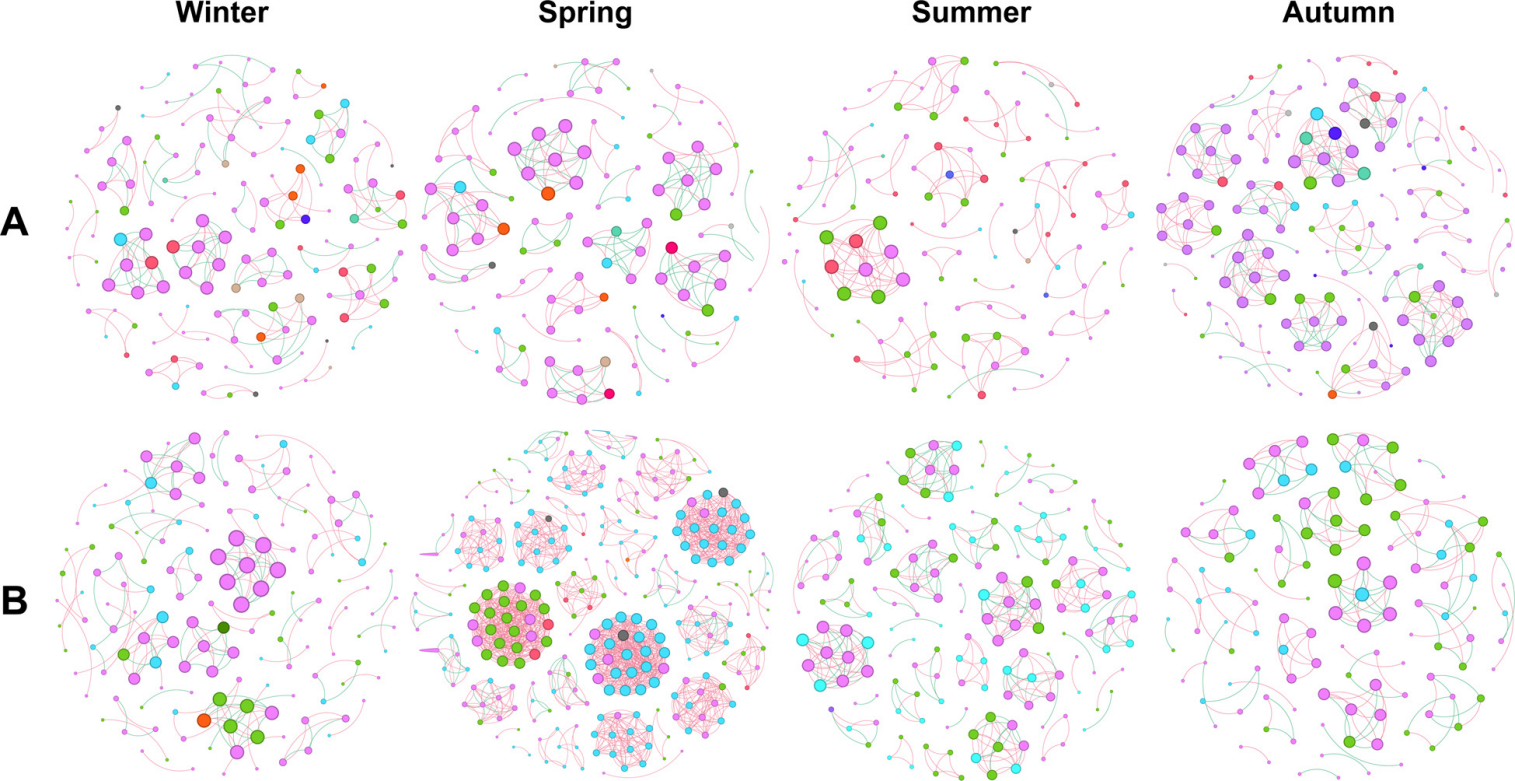
Co-occurrence network analysis of the bacterial **(A)** and fungal **(B)** community associated with pomelo leaves in each season. The different colors in each network indicate different phyla of bacteria **(A)** or fungi **(B)**.

To further explore the difference in phyllosphere microbiome across seasons, we performed network analysis of bacterial or fungal community in each season. The lowest values of node number, edge number, modularity were observed in summer and the highest values were observed in spring or autumn, for bacterial community; while the lowest values in node number, edge number, average degree, network density, and modularity were observed in summer, autumn or winter, and the highest values were observed in spring for fungal community (**Figure 6**, **Table 2**). This confirms the differential annual patterns between bacterial and fungal community of pomelo leaves as revealed in **Figure 5**.

**Table 2:** The network properties of microbial community on the pomelo leaves sampled across annual cycle.

Network properties	Winter (Dec. 2022)	Spring (Feb. 2023)	Summer (May 2023)	Autumn (Aug. 2023)
Bacterial community				
Node number	143	120	113	185
Edge number	189	200	141	164
Average degree	2.643	3.333	2.496	1.773
Network density	0.019	0.028	0.022	0.010
Modularity	0.949	0.927	0.908	0.977
Fungal community				
Node number	149	280	167	119
Edge number	190	1362	340	177
Average degree	2.550	9.729	4.072	2.975
Network density	0.017	0.035	0.025	0.025
Modularity	0.939	0.984	0.937	0.940

According to beta diversity, it is clear that phyllosphere micobiome were strongly shaped by seasonality, which was closely associated with weather parameters. We probed into the annual dynamics of precipitation and air temperature, and found that winter and spring were characteristic of low precipitation and air temperature, while summer and autumn were characteristic of high precipitation and air temperature (**Figure 1**).

**Figure 7:**
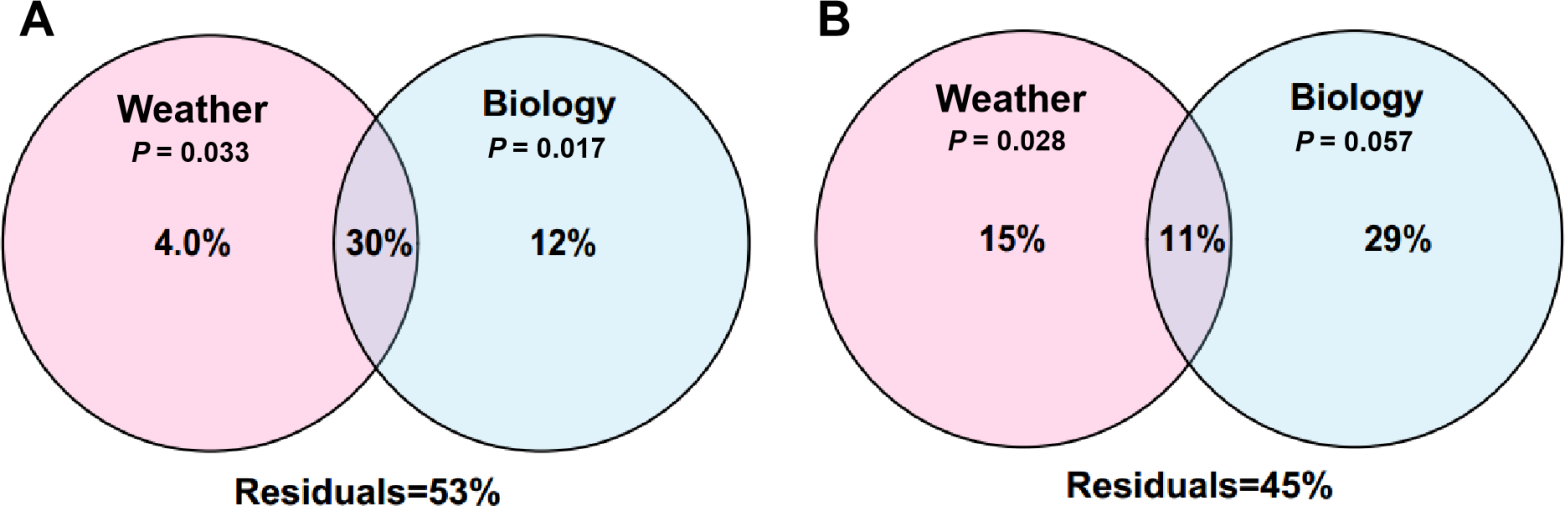
Variation partitioning analysis (VPA) showing the respective contributions of weather parameters and biological factors to bacterial **(A)** and fungal **(B)** community assembly.

Considering the effects of biological factors (leaf traits) on phyllosphere microbiome (Li et al., 2022a; Yuan et al., 2023), we further measured 24 leaf nutritional constituents, including 16 amino acids, 2 carbohydrates, and 6 nutrients (**Supplementary Data Sheet S2**). Then we performed VPA to compare the effect size of weather parameters and biological factors. Leaf traits contributed 12% of the variation in bacterial community, much higher than climate (4%). Meanwhile, they had an overlap of 30%, indicating a strong interplay between climate and leaf traits (**Figure 7**). For fungal community, leaf traits and climate contributed 29% and 15%, respectively, with an interplay of 11% (**Figure 7**). In general, it seems that leaf traits exerted a greater effect on phyllosphere microbiome of pomelo than climate.

### 3.4 Phyllosphere bacterial and fungal community shaped by biological factors

**Figure 8:**
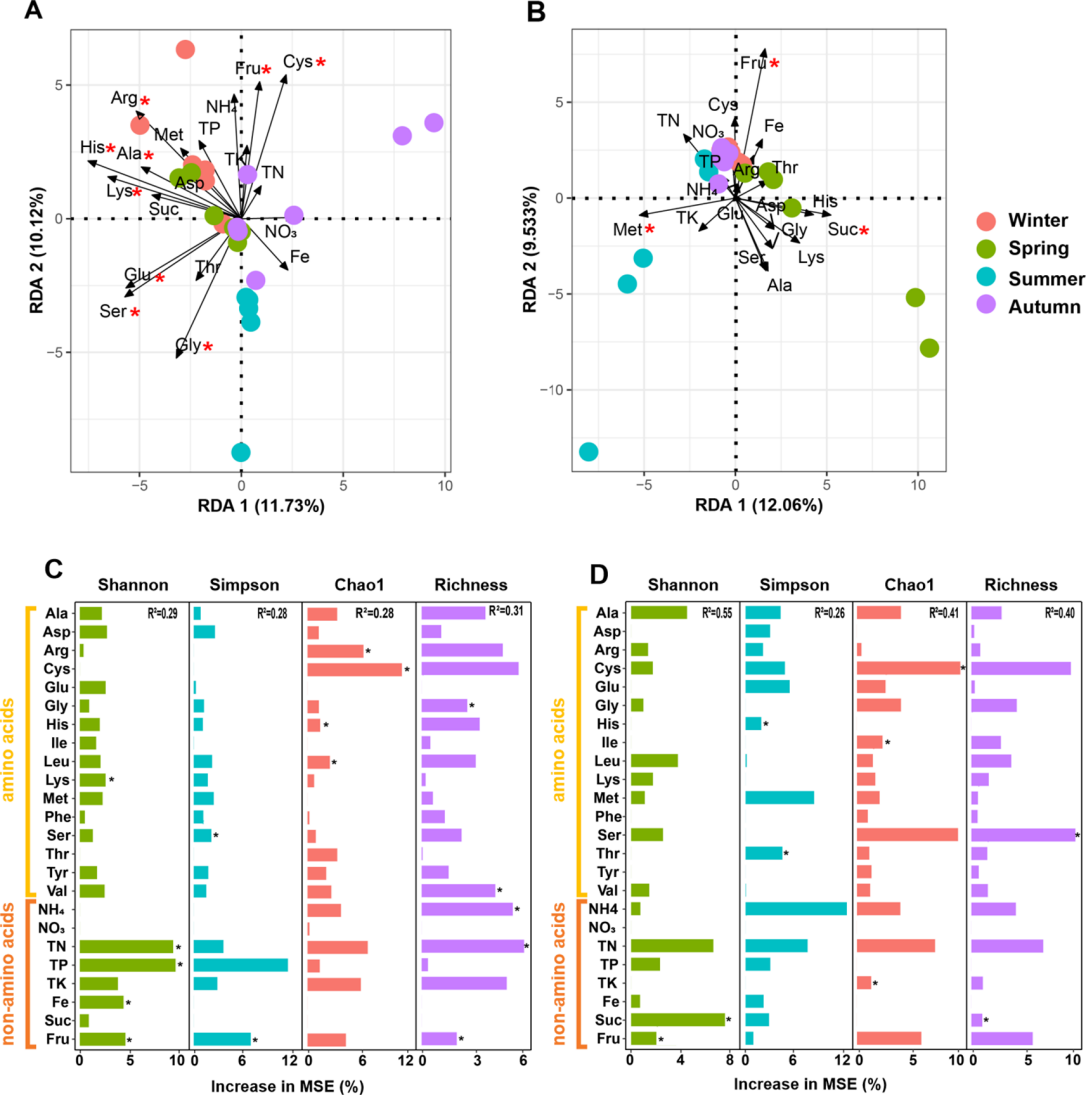
Redundancy analysis revealing the effects of leaf traits on the bacterial **(A, B)** and fungal **(C, D)** community associated with pomelo leaves. **(B, D)** indicate the quantitative effect of each leaf trait. Red aristers in **(A, B)** indicate significant influences. Black aristers in **(C, D)** indicate significant effects.

**Figure 9:**
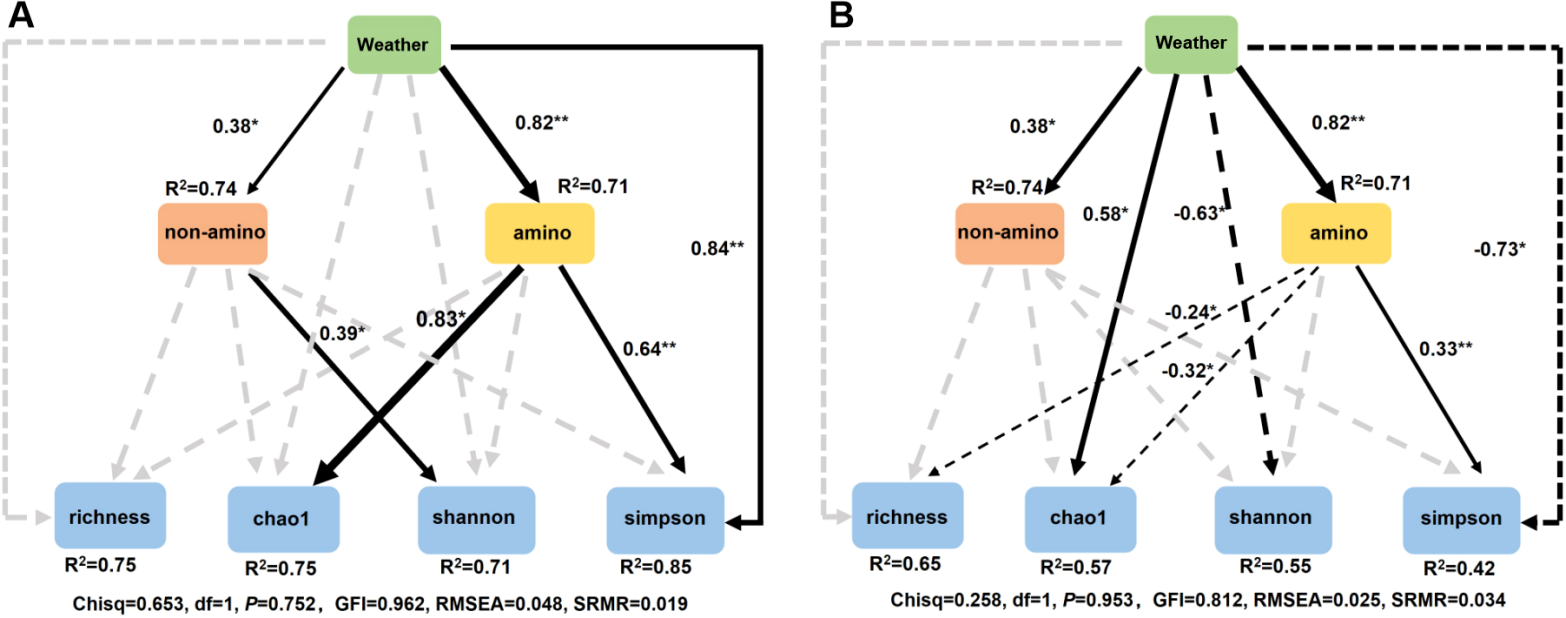
Structure equation model (SEM) analysis integrating the effects of weather parameters and biological factors on alpha diversity of bacterial **(A)** and fungal **(B)** community.

Since leaf traits were more effective in shaping phyllosphere microbiome than climate, we focused on these nutritional constituents. Three rounds of collinearity analysis revealed that His was collinear with Leu, Ile, and Val, and Lys was collinear with Tyr and Phe (**Supplementary Figure S1**). Therefore, Leu, Ile, Val, Tyr, and Phe were excluded but only 19 nutritional constituents entered the following RDA. RDA revealed that 6 kinds of amino acids significantly shaped bacterial community, with Lys, Arg, and Ser ranking the top three, however, only 2 non-amino acid parameters (Fru and Glu) exerted significant influence (**Figure 8A**). For fungal community, only 3 constituents, including Fru, Met, and Suc exerted significant influences (**Figure 8B**). Similarly, RF analysis revealed that 8 kinds of amino acids and 5 non-amino acid constituents significantly affected the alpha diversity of bacterial community, while 5 kinds of amino acids and 3 non-amino acid constituents significantly affected the alpha diversity of fungal community (**Figures 8C, D**). SEM demonstrates that climate showed a positive effect on both the amino acids and the non-amino acid constitutes in leaves, which further positively affected the alpha diversity of bacterial community. In contrast, non-amino acid constitutes did not affected the alpha diversity of fungal community, but climate directly affected it (**Figure 9**). This indicates the more complicated influences of climate and leaf traits on fungal community than on bacterial community.

## 4 Discussion

Bacterial and fungal communities colonizing phyllosphere are critical components of plant microbiome, which play a essential role in maintaining plant health, nutrient acquisition and stress resistance (e.g. N) (Chen et al., 2020; Li et al., 2022b; Zhu et al., 2023; Li et al., 2024). However, phylosphere microbiome has been less explored so far compared to rhizosphere microbiome. Phyllosphere microbiome is highly dynamic in response to environmental cues, which include both biotic and abiotic factors (Thapa et al., 2017; Li et al., 2023; Wang et al., 2023; Kong et al., 2024). In this study, we demonstrate that both weather parameters (precipitation and air temperature) and biological factors (leaf traits) shaped the bacterial and fungal communities of pomelo leaves, with plant factors exerting a greater influence. This is similar to the results by Zhou et al. (2023), who indicated that environmental factors (geographic location and climatic conditions) and host genotype affected the epiphytic bacterial and fungal communities of wild soybeans across China. However, plant traits contributed 12%-19% to the variation of microbial community, much higher than weather parameters (4%-15%) in our study, while environmental factors contributed 19.9%-25.8% to the variation, much higher than host genotype (0.4%-3.6%) in the study by Zhou et al. (2023). This suggests that the relative importance of environmental factors and plant factors might depend on context, such as plant species, sampling area. It is well established that plant microbiome can be regulated both directly by plant traits (internal factors) and indirectly by environments (external factors) (Li et al., 2023; Kong et al., 2024), and external factors normally work via their influences on internal factors. The seasonal fluctuations of amino acids and sugars in citrus leaves have been reported, normally with low contents in the actively growing seasons (e.g. summer) (Yildiz et al., 2013; Xiong et al., 2024). Additionally, plant leaves can release volatile organic compounds (VOCs), which might possess antimicrobial activity and act as carbon sources, thereby regulating phyllosphere microbiome (Farré-Armengol et al., 2016). Specifically, *Citrus* plants are well recognized for their fragrance (namely VOCs), which was demonstrated to strongly structure their phyllosphere bacterial community (Wang et al., 2022). In this study, it is possible that the weather parameters greatly affected the plant traits (such as amino acids and sugars in leaves), especially in summer when the vegetative growth of pomelo plants was vigorous with both high temperature and high precipitation. Moreover, the VOC profile of *Citrus* plants varies much depending on seasonality (Lin et al., 2022), thereby probably contributing to the seasonal pattern of phyllosphere microbiome in this study. Considering the coupled effects of appropriate climate conditions and N fertilizers in promoting plant vegetative growth, N fertilizer application is necessary to regulate the phyllosphere microbiome even with appropriate temperature and precipitation in citrus production systems.

It is interesting that the contribution of weather parameters to bacterial community (4.0%) was much lower than that to fungal community (15%). It is possible that fungal community is more sensitive to weather parameters, especially to environmental moisture (monitored as precipitation in this study) than bacterial community (Kaisermann et al., 2015; He et al., 2023). Moreover, it is notable that the overlap of weather parameters and biological factors for bacterial community (30%) was much higher than that for fungal community (11%). This suggests that the influence of weather parameters on the bacterial community was more likely dependant on their effects on plant traits, while weather parameters influenced the fungal community in a relatively independant manner.

The core taxa of microbiome are defined as the members shared by all or most microbial communities with similar backgrounds, and play essential roles in the community functioning (Shade and Handelsman, 2012; Ren and Wu, 2016). For example, Shen et al. (2022) demonstrated that the core taxa (mainly belonging to Myxococcales, Pseudomonadales, Xanthomonadales) of suppressive soils from six banana plantation sites showed protective effects against banana *Fusarium* wilt disease, compared to the core taxa of conducive soils. In this study, we explored the core taxa of phyllosphere microbiome according to occupancy, and identified *Methylobacterium-Methylorubrum*, *Pseudomonas*, *Hymenobacter*, *Sphingomonas*, and *Massilia* as the top 5 core genera of bacterial community for pomelo, among which *Methylobacterium-Methylorubrum*, *Pseudomonas*, and *Sphingomonas* were also the core genera of wild soybean (Zhou et al., 2023). *Methylobacterium-Methylorubrum* is one of the most commonly reported phyllosphere bacteria promoting growth performance of many plant species, such as cucumber (Zhang et al., 2025) and rice (Oeum et al., 2024), which has been intensively investigated regarding its colonization capacity and functionality (Abanda-Nkpwatt et al., 2006; Yurimoto et al., 2021). The mechanisms underlying the plant growth promotion by *Methylobacterium-Methylorubrum* in phyllosphere mainly include nitrogen fixation, secretion of auxin, cytokinin, and 1-aminocyclopropane-1-carboxylate deaminase, and etc (Zhang et al., 2021). Recently, Zhang et al. (2024) demonstrated that the phosphoribosylpyrophosphate synthetase of *Methylorubrum extorquens* AM1 facilitated its superior colonization capability and functionality. It is possible that *Methylobacterium-Methylorubrum* bacteria assimilate methanol emitted from phyllosphere and then provide carbon sources to other members in the community. *Pseudomonas* is frequently recognized as beneficial member of phyllosphere community. Li et al. (2025) inoculated *P. fluorescens* to *Salix matsudana* and showed a increase of 90.51% in plant biomass. In detail, inoculation increased the asymbiotic nitrogen-fixation, improved photosynthetic traits (e.g. net photosynthetic rate, intercellular CO_2_ concentration, stomatal conductance, transpiration rate) and the root traits (e.g. root length, root branching) and modified the phyllosphere microbiome beneficial for plant health, thereby promoting the plant nutrient uptake and biomass. Su et al. (2024) indicated that the compound 4-hydroxycinnamic acid synthesized by OsPAL02 in rice plants enriched Pseudomonadales in phyllosphere, while the reduced Pseudomonadales abundance in the knockout mutant of *OsPAL02* resulted in the dysbiosis of phyllosphere microbiome and higher susceptibility to disease. These studies suggest that *Pseudomonas*, either native or inoculated, might function via maintaining homeostasis of phyllosphere microbiome in most cases.

Our study reveals that *Acrodontium*, *Hyphozyma*, *Inocybe* were the top 3 core fungal genera of pomelo leaves. *Acrodontium* is the frequent colonizer of citrus leaves, which was enriched in healthy trees compared to HLB-infected trees and thus was regarded as the keystone taxa of phyllosphere fungal microbiome (Ginnan et al., 2020). It is interesting that a *Hyphozyma* species (*H. roseoniger*) can convert sclareol to ambradiol (Ncube et al., 2022), which might contribute to the production of fragrance compounds of pomelo leaves. Surprisingly, however, *Inocybe* has been frequently reported as dominant and ectomycorrhizal fungus (Nara, 2009; Bohorquez et al., 2021; Khan and Reshi, 2022), occasionally occurring in leaf litter (Liber et al., 2022).

The chemical properties of plant leaves are primary factors shaping phyllosphere microbiome (Yadav et al., 2005; Li et al., 2022a; Luo et al., 2023). When nutrient contents such as N, P, K and their stoichiometry have been explored for a long time, in this study, we focused on the amino acids in leaves and found that amino acids contributed much to the variation in microbial community, which has been less reported before. Our previous work on rhizosphere microbiome revealed that amino acids greatly regulated bacterial community in rhizosphere, and phenylalanine was the most effective in promoting soil N cycling (Feng et al., 2021; Feng et al., 2023a). This study reveals that amino acids played much more important roles in shaping bacterial community than non-amino acid constituents, such as several kinds of mineral nutrients, with Lys, Arg, and Ser more effective than others. Our results put the special importance on the amino acids in leaves for the first time although other chemical properties of leaves have been investigated. This importance can be attributed to the fact that amino acids can provide both N and C sources to the phyllosphere microbiome (Feng et al., 2021, 2023a). Similarly, several studies also shed lights on amino acids. Proline, tyrosine, serine and phenylalanine showed important influence on the phyllosphere microbiome of nettle (*Urtica cannabina*), with the affected taxa including both bacteria (*Enterococcus*, *Hymenobacter*, *Sphingomonas*, *Sphingobacterium*, *Massilia*, *Ochrobactrum*, Oxalobacteraceae) and fungi (*Pezizella*, *Udeniomyces*, *Filobasidium*, Didymellaceae, Glomerellales, Helotiales) (Jia et al., 2023). Total fee AAs were one of the most outstandingly determined factors interacting with phyllosphere microbiome of garden lettuce, with the functional taxa (*Kinetoplastibacterium*, *Natronococcus*, *Bacillus*, *Bradyrhizobium*, *Methanococcus*) harboring *mdh* or *glyA* genes significantly affected (Kong et al., 2024). In contrast, fungal community (e.g. *Taphrina*, *Cylindrocladiella*, *Aspergillus*, *Boletus*, *Malassezia*, *Cladosporium*, *Xenocylindrocladium*, *Cordyceps*, *Pyrenochaeta*) in the phyllosphere of tea plants was more sensitive to sugars than bacterial community (Chen et al., 2024), which might be due to their differential trophism. Since the phyllosphere microbime is significantly regulated by the amino acids and sugars in leaves, the future research can focus on the fine regulatory patterns of these compounds on some specific taxa, such as *Methylobacterium-Methylorubrum*, *Pseudomonas*, *Sphingomonas*, which represent beneficial taxa for plant performance (Li et al., 2022b, 2024; Zhang et al., 2024). As such, it is possible to develop amino acid-based biostimulants enriching the beneficial phyllosphere microbial taxa, which supports the sustainability of agricultural production.

## 5 Conclusion

Phyllosphere microbime can promote plant disease resistance and growth performance. Therefore, the understanding of the annual dynamics and drivers of phyllosphere microbime is pivotal to the utilization of it. Therefore, we characterized the phyllosphere bacterial and fungal communities across annual cycle, and identified *Methylobacterium-Methylorubrum*, *Pseudomonas*, *Hymenobacter* and *Acrodontium*, *Hyphozyma*, *Inocybe* as the top core taxa of pomelo phyllosphere microbiome. The bacterial community in summer and the fungal community in autumn were much different from those in other seasons, respectively. Both biological factors (including 24 leaf traits) and weather parameters (temperature and precipitation) affected microbiome assembly, with the former (12%-29%) contributing more to the assemblage than the latter (4%-15%). Furthermore, we demonstrated for the first time that amino acids and sugars in leaves were the main drivers of bacterial and fungal communities, respectively, highlighting the importance of amino acids in manipulating phyllosphere microbiome. In general, our data suggest that biological factors (e.g. amino acids and sugars in leaves) and weather parameters regulate the phyllosphere microbiome in direct and indirect ways, respectively.

## Data Availability

The datasets presented in this study can be found in online repositories. The names of the repository/repositories and accession number(s) can be found in the article/**Supplementary Material**.
