# Supplementary material for: Weather parameters and biotic factors synergistically shape the phyllosphere microbiome of pomelo (Citrus maxima (Burm.) Merr.) across annual cycle
Source: Front Plant Sci. 2025 Apr 3;16:1532188. doi: 10.3389/fpls.2025.1532188 (PMC12003388; doi:10.3389/fpls.2025.1532188)

**Figure S1.** Colinearity analysis of 24 leaf traits. A: the first round of colinearity analysis revealed the colinearity of His, Leu, Ile, and Val, and the colinearity of Phe, Lys, and Tyr. B: The second round of colinearity analysis revealed the colinearity of Phe and Lys. C: the third round of colinearity analysis.

###
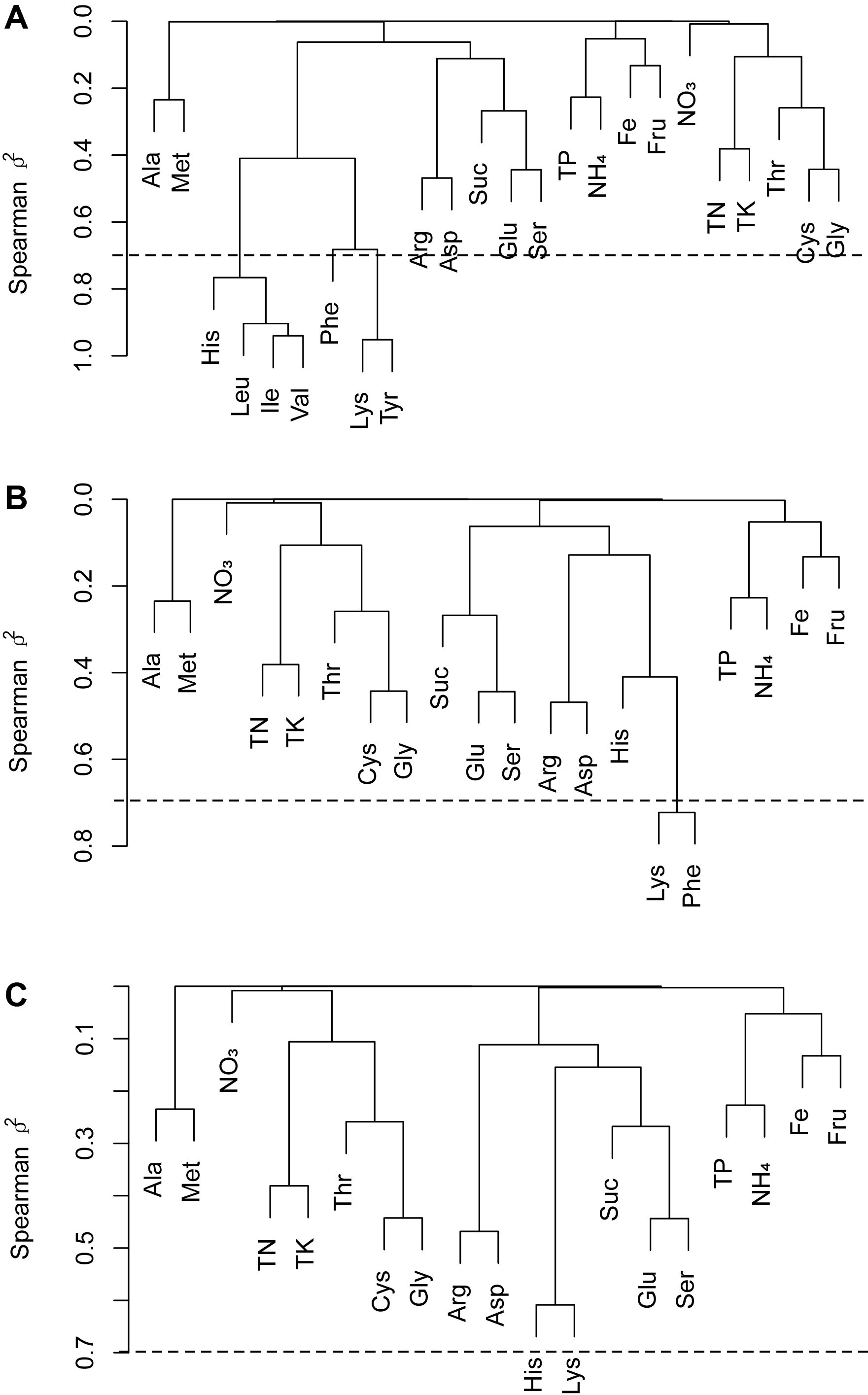

Supplement: Supplementary file 1 [file Table1.docx]
